# *cis*-Difluoridobis(1,10-phenanthroline)chromium(III) perchlorate monohydrate

**DOI:** 10.1107/S1600536808001153

**Published:** 2008-01-16

**Authors:** Torben Birk, Jesper Bendix, Högni Weihe

**Affiliations:** a Department of Chemistry, University of Copenhagen, Universitetsparken 5, DK-2100 København Ø, Denmark

## Abstract

The title complex, [CrF_2_(C_12_H_8_N_2_)_2_]ClO_4_·H_2_O, displays a slightly distorted octa­hedral coordination geometry around the central chromium(III) ion. The Cr environment is composed of a *cis* arrangement of two 1,10-phenanthroline [average Cr^III^—N = 2.0726 (10) Å] and two fluoride [average Cr^III^—F = 1.8533 (6) Å] ligands. The water molecule forms a hydrogen bond to fluorine in a neighbouring cation.

## Related literature

For details of the general synthesis of amine-containing difluorido complexes of chromium(III), see: Glerup *et al.* (1970 ▶). For the structure of the analogous 2,2′-bipyridine complex, see: Yamaguchi-Terasaki *et al.* (2007 ▶). For related literature, see: Brenčič *et al.* (1981 ▶, 1987 ▶); Delavar & Staples (1981 ▶); Kaizaki & Takemoto (1990 ▶); Kane-Maguire *et al.* (1986 ▶).
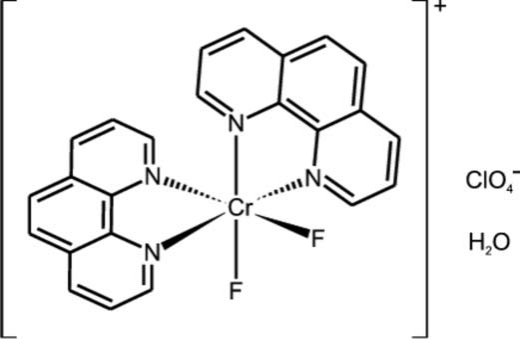


## Experimental

### 

#### Crystal data

[CrF_2_(C_12_H_8_N_2_)_2_]ClO_4_·H_2_O
*M* *_r_* = 567.87
Triclinic, 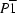

*a* = 7.6930 (10) Å
*b* = 9.4640 (8) Å
*c* = 16.0610 (17) Å
α = 79.750 (7)°
β = 83.228 (12)°
γ = 88.115 (8)°
*V* = 1142.6 (2) Å^3^
*Z* = 2
Mo *K*α radiation
μ = 0.68 mm^−1^
*T* = 122 (1) K
0.44 × 0.41 × 0.16 mm

#### Data collection

Nonius KappaCCD area-detector diffractometer
Absorption correction: Gaussian integration (Coppens, 1970 ▶) *T*_min_ = 0.794, *T*_max_ = 0.913
28606 measured reflections
4014 independent reflections
3851 reflections with *I* > 2σ(*I*)
*R*_int_ = 0.025

#### Refinement

*R*[*F* ^2^ > 2σ(*F* ^2^)] = 0.031
*wR*(*F* ^2^) = 0.145
*S* = 1.41
4014 reflections
329 parameters
H-atom parameters constrained
Δρ_max_ = 0.79 e Å^−3^
Δρ_min_ = −0.51 e Å^−3^

### 

Data collection: *COLLECT* (Nonius, 1999 ▶); cell refinement: *COLLECT*; data reduction: *EvalCCD* (Duisenberg *et al.*, 2003 ▶); program(s) used to solve structure: *SHELXS97* (Sheldrick, 2008 ▶); program(s) used to refine structure: *SHELXL97* (Sheldrick, 2008 ▶); molecular graphics: *ORTEP-3* (Farrugia, 1997 ▶); software used to prepare material for publication: *SHELXL97*.

## Supplementary Material

Crystal structure: contains datablocks global, I. DOI: 10.1107/S1600536808001153/wk2075sup1.cif

Structure factors: contains datablocks I. DOI: 10.1107/S1600536808001153/wk2075Isup2.hkl

Additional supplementary materials:  crystallographic information; 3D view; checkCIF report

## Figures and Tables

**Table:** 

Cr1—F2	1.8444 (10)
Cr1—F1	1.8621 (10)
Cr1—N4	2.0566 (15)
Cr1—N2	2.0607 (15)
Cr1—N3	2.0797 (16)
Cr1—N1	2.0934 (15)

**Table:** 

F2—Cr1—F1	95.92 (5)
F2—Cr1—N4	92.33 (5)
F1—Cr1—N4	91.42 (5)
F2—Cr1—N2	91.83 (6)
F1—Cr1—N2	91.86 (5)
N4—Cr1—N2	174.40 (5)
F2—Cr1—N3	89.38 (5)
F1—Cr1—N3	170.08 (5)
N4—Cr1—N3	79.95 (6)
N2—Cr1—N3	96.36 (6)
F2—Cr1—N1	170.54 (5)
F1—Cr1—N1	88.67 (5)
N4—Cr1—N1	95.83 (6)
N2—Cr1—N1	79.72 (6)
N3—Cr1—N1	87.34 (6)

**Table 2:** Hydrogen-bond geometry (Å, °)

*D*—H⋯*A*	*D*—H	H⋯*A*	*D*⋯*A*	*D*—H⋯*A*
O5—H5*B*⋯F1^i^	1.03	1.69	2.7183 (19)	175

Symmetry code: (i) 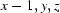.

**Fig. 1.**
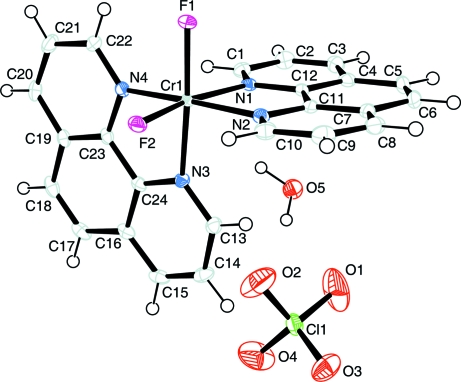
The molecular structure and atom labeling scheme of cis-[Cr(phen)2F2]ClO4. H2O. Displacement ellipsoids are drawn at 50% probability. H atoms with arbitrary radii.

**Fig. 2.**
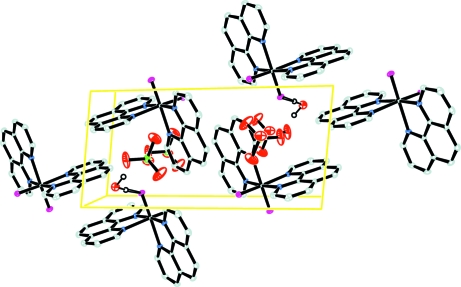
The crystal packing in cis-[Cr(phen)2F2]ClO4. H2O. Displacement ellipsoids are drawn at 50% probability. H atoms except the one originated from cystal water have been omitted.

## supplementary crystallographic information

### Comment

Difluoro complexes of chromium(III) with various amine ligands have received a steady interest in the literature. Areas of interest have been *e.g.* kinetic behavior (Delavar & Staples, 1981), solvatochromism (Kaizaki & Takemoto, 1990) and photochemical/photophysical properties (Kane-Maguire *et al.*, 1986). From a synthetic point of view simple fluoro containing complexes exhibit some advantageous properties for synthesis in non-acidic media. The strong coordination of the small and basic fluoro ligand makes it suitable as an "inorganic" protection group, easily removed and substituted by other ligands. Only a limited number of complexes belonging to this group have been structural characterized *e.g. cis*-[Cr(NH_3_)_4_F_2_]ClO_4_ (Brenčič *et al.*, 1981), *cis*-[Cr(en)_2_F_2_]ClO_4_^.^ NaClOO_4_^.^ H_2_O (Brenčič *et al.*, 1987) and *cis*-[Cr(bipy)_2_F_2_]ClO_4_ (Yamaguchi-Terasaki *et al.*, 2007). In this report we present the crystal structure of *cis*-Difluoro(1,10-phenanthroline)chromium(III) perchlorate monohydrate (1).

The structure of (1) shows a octahedral coordination geometry around the central chromium(III) ion consisting of a *cis* arrangment of two fluorine and two nitrogen ligator atoms (Figure 1). Comparison of the Cr—N bond distances in *trans* position relative to the fluoro ligand [N_1_: 2.0934 (15) Å and N_3_: 2.0797 (16) Å] show a slightly elongation compared to the corresponding in *cis* postion [N_2_: 2.0607 (15) Å and N_4_: 2.0566 (15) Å]. This pattern of bond lengths are similar to that found in the analogous bipyridine complex *cis*-Difluoro(2,2'-bipyridine)chromium(III) perchlorate, *cis*-[Cr(bipy)_2_F_2_]ClO_4_.

The overall crystal packing is predominately determined by the approximately perpendicular orientation of the two planar 1,10-phenanthroline ligands [N_3_—Cr—N_1_:87.34 (6) °, N_3_—Cr—N_2_: 96.36 (6) °] and the presence of crystal water connecting each asymmetric unit with another through hydrogen bonding from water to fluorine (Figure 2).

### Experimental

The title complex was synthesized by reflux of *trans*-difluorotetrakis(pyridine)chromium(III) perchlorate and 1,10-phenanthroline in 2-methoxyethanol according to the published method (Glerup *et al.*, 1970).

Crystal suitable for X-ray diffraction were obtained by the following method: 0.208 g of the compound was dissolved in a solution of water/acetonitrile (20 ml/10 ml) and filtered though a filter paper into a small beaker. The beaker was covered with a lid of paper and left undisturbed at room temperature for crystallization (*ca* 3–5 days). The crystals was harvested by gently scratching with a spatula and washed with the mother liquid.

### Refinement

All H atoms were identified in a difference Fourier map and incorporated in the refinement in a riding model, with C–H = 0.95 Å and *U*_iso_(H) = 1.2*U*_Eq_.

### Crystal data

**Table:**

[CrF_2_(C_12_H_8_N_2_)_2_]ClO_4_·H_2_O	*Z* = 2
*M**_r_* = 567.87	*F*_000_ = 578
Triclinic, *P*1	*D*_x_ = 1.651 Mg m^−^^3^
Hall symbol: -P 1	Mo *K*α radiation λ = 0.71073 Å
*a* = 7.6930 (10) Å	Cell parameters from 26598 reflections
*b* = 9.4640 (8) Å	θ = 2.3–25.0º
*c* = 16.0610 (17) Å	µ = 0.68 mm^−^^1^
α = 79.750 (7)º	*T* = 122 (1) K
β = 83.228 (12)º	Block, red
γ = 88.115 (8)º	0.44 × 0.41 × 0.16 mm
*V* = 1142.6 (2) Å^3^	

### Data collection

**Table:**

Nonius KappaCCD area-detector diffractometer	4014 independent reflections
Radiation source: fine-focus sealed tube	3851 reflections with *I* > 2σ(*I*)
Monochromator: graphite	*R*_int_ = 0.025
*T* = 122.0(10) K	θ_max_ = 25.0º
ω and φ scans	θ_min_ = 2.3º
Absorption correction: gaussian integration(Coppens, 1970)	*h* = −9→9
*T*_min_ = 0.794, *T*_max_ = 0.913	*k* = −11→11
28606 measured reflections	*l* = −18→19

### Refinement

**Table:**

Refinement on *F*^2^	H-atom parameters constrained
Least-squares matrix: full	*w* = 1/[σ^2^(*F*_o_^2^) + (0.1*P*)^2^] where *P* = (*F*_o_^2^ + 2*F*_c_^2^)/3
*R*[*F*^2^ > 2σ(*F*^2^)] = 0.031	(Δ/σ)_max_ = 0.078
*wR*(*F*^2^) = 0.145	Δρ_max_ = 0.79 e Å^−^^3^
*S* = 1.41	Δρ_min_ = −0.51 e Å^−^^3^
4014 reflections	Extinction correction: none
329 parameters	

### Special details

**Table:**

Geometry. All e.s.d.'s (except the e.s.d. in the dihedral angle between two l.s. planes) are estimated using the full covariance matrix. The cell e.s.d.'s are taken into account individually in the estimation of e.s.d.'s in distances, angles and torsion angles; correlations between e.s.d.'s in cell parameters are only used when they are defined by crystal symmetry. An approximate (isotropic) treatment of cell e.s.d.'s is used for estimating e.s.d.'s involving l.s. planes.
Refinement. Refinement of *F*^2^ against ALL reflections. The weighted *R*-factor *wR* and goodness of fit *S* are based on *F*^2^, conventional *R*-factors *R* are based on *F*, with *F* set to zero for negative *F*^2^. The threshold expression of *F*^2^ > σ(*F*^2^) is used only for calculating *R*-factors(gt) *etc*. and is not relevant to the choice of reflections for refinement. *R*-factors based on *F*^2^ are statistically about twice as large as those based on *F*, and *R*- factors based on ALL data will be even larger.

### Fractional atomic coordinates and isotropic or equivalent isotropic displacement parameters (Å^2^)

**Table:**

	*x*	*y*	*z*	*U*_iso_*/*U*_eq_	
Cr1	0.86046 (3)	0.55137 (3)	0.266315 (15)	0.01130 (17)	
Cl1	0.42169 (6)	0.04581 (5)	0.27329 (3)	0.02027 (19)	
F1	1.08731 (13)	0.60734 (11)	0.22192 (6)	0.0170 (3)	
F2	0.92467 (13)	0.41293 (11)	0.35383 (6)	0.0175 (3)	
N1	0.76931 (19)	0.68048 (16)	0.16003 (9)	0.0124 (3)	
N3	0.6003 (2)	0.52586 (16)	0.31812 (9)	0.0136 (3)	
N4	0.83917 (19)	0.70717 (16)	0.34149 (9)	0.0146 (3)	
N2	0.8597 (2)	0.40348 (16)	0.18644 (10)	0.0144 (3)	
C12	0.7592 (2)	0.60779 (19)	0.09479 (11)	0.0137 (4)	
C1	0.7256 (2)	0.81930 (19)	0.14890 (12)	0.0171 (4)	
H1	0.7315	0.8707	0.1942	0.020*	
C24	0.5533 (2)	0.61063 (18)	0.37709 (10)	0.0138 (4)	
C23	0.6826 (2)	0.70727 (19)	0.39112 (10)	0.0139 (4)	
C13	0.4814 (2)	0.43556 (19)	0.30366 (12)	0.0173 (4)	
H13	0.5134	0.3751	0.2629	0.021*	
C15	0.2655 (2)	0.5115 (2)	0.40692 (12)	0.0197 (4)	
H15	0.1510	0.5056	0.4367	0.024*	
C11	0.8100 (2)	0.45883 (19)	0.10871 (11)	0.0140 (4)	
C10	0.9151 (2)	0.26736 (19)	0.20079 (12)	0.0191 (4)	
H10	0.9493	0.2277	0.2551	0.023*	
C18	0.4782 (3)	0.7862 (2)	0.50181 (12)	0.0202 (4)	
H18	0.4531	0.8434	0.5448	0.024*	
C22	0.9635 (2)	0.7957 (2)	0.35158 (12)	0.0183 (4)	
H22	1.0730	0.7964	0.3173	0.022*	
C7	0.8106 (2)	0.3795 (2)	0.04286 (12)	0.0171 (4)	
C19	0.6457 (2)	0.79451 (19)	0.45241 (11)	0.0164 (4)	
C9	0.9245 (3)	0.1812 (2)	0.13812 (13)	0.0224 (4)	
H9	0.9671	0.0851	0.1497	0.027*	
C20	0.7809 (3)	0.8874 (2)	0.46210 (12)	0.0208 (4)	
H20	0.7628	0.9482	0.5035	0.025*	
C8	0.8722 (3)	0.2354 (2)	0.05988 (13)	0.0212 (4)	
H8	0.8771	0.1771	0.0173	0.025*	
C3	0.6631 (2)	0.8201 (2)	0.00597 (12)	0.0204 (4)	
H3	0.6278	0.8691	−0.0464	0.024*	
C21	0.9360 (3)	0.8881 (2)	0.41136 (13)	0.0231 (4)	
H21	1.0260	0.9512	0.4168	0.028*	
C17	0.3534 (2)	0.6962 (2)	0.48787 (12)	0.0201 (4)	
H17	0.2421	0.6933	0.5207	0.024*	
C2	0.6715 (3)	0.8918 (2)	0.07198 (12)	0.0214 (4)	
H2	0.6406	0.9908	0.0661	0.026*	
C6	0.7569 (2)	0.4481 (2)	−0.03680 (12)	0.0198 (4)	
H6	0.7560	0.3946	−0.0815	0.024*	
C5	0.7068 (3)	0.5882 (2)	−0.05005 (12)	0.0205 (4)	
H5	0.6709	0.6311	−0.1035	0.025*	
C4	0.7071 (2)	0.6730 (2)	0.01613 (11)	0.0168 (4)	
C16	0.3878 (2)	0.6065 (2)	0.42455 (11)	0.0164 (4)	
C14	0.3132 (2)	0.4267 (2)	0.34592 (12)	0.0205 (4)	
H14	0.2312	0.3630	0.3333	0.025*	
O3	0.3942 (2)	−0.10036 (16)	0.26532 (11)	0.0357 (4)	
O2	0.5918 (3)	0.0563 (2)	0.29920 (14)	0.0537 (5)	
O4	0.2950 (3)	0.0879 (2)	0.33573 (14)	0.0617 (7)	
O1	0.4169 (4)	0.1381 (2)	0.19353 (12)	0.0641 (7)	
O5	0.1662 (2)	0.87161 (16)	0.12962 (9)	0.0286 (4)*	
H5A	0.2568	0.9069	0.1647	0.034*	
H5B	0.1306	0.7742	0.1664	0.034*	

### Atomic displacement parameters (Å^2^)

**Table:**

	*U*^11^	*U*^22^	*U*^33^	*U*^12^	*U*^13^	*U*^23^
Cr1	0.0102 (2)	0.0141 (2)	0.0097 (2)	−0.00101 (14)	0.00002 (14)	−0.00270 (15)
Cl1	0.0265 (3)	0.0206 (3)	0.0139 (3)	0.0005 (2)	−0.0013 (2)	−0.0043 (2)
F1	0.0123 (5)	0.0210 (6)	0.0168 (6)	−0.0029 (4)	0.0026 (4)	−0.0030 (4)
F2	0.0128 (5)	0.0227 (6)	0.0152 (5)	−0.0009 (4)	−0.0013 (4)	0.0017 (4)
N1	0.0098 (7)	0.0129 (7)	0.0140 (7)	−0.0007 (6)	0.0019 (6)	−0.0029 (6)
N3	0.0110 (7)	0.0157 (7)	0.0139 (7)	−0.0005 (6)	−0.0028 (5)	−0.0011 (6)
N4	0.0136 (8)	0.0185 (8)	0.0117 (7)	−0.0003 (6)	−0.0011 (6)	−0.0024 (6)
N2	0.0140 (8)	0.0136 (8)	0.0154 (8)	−0.0016 (6)	0.0019 (6)	−0.0038 (6)
C12	0.0090 (8)	0.0195 (9)	0.0125 (9)	−0.0024 (7)	0.0010 (7)	−0.0038 (7)
C1	0.0124 (9)	0.0166 (9)	0.0224 (10)	0.0005 (7)	−0.0003 (7)	−0.0051 (7)
C24	0.0128 (9)	0.0153 (8)	0.0114 (8)	0.0024 (7)	−0.0023 (6)	0.0027 (7)
C23	0.0146 (9)	0.0161 (8)	0.0107 (8)	0.0029 (7)	−0.0030 (7)	−0.0010 (7)
C13	0.0182 (9)	0.0174 (9)	0.0164 (9)	−0.0010 (7)	−0.0055 (7)	−0.0008 (7)
C15	0.0110 (9)	0.0219 (9)	0.0217 (10)	0.0009 (7)	−0.0006 (7)	0.0073 (7)
C11	0.0102 (8)	0.0181 (9)	0.0139 (9)	−0.0024 (7)	0.0015 (7)	−0.0046 (7)
C10	0.0183 (9)	0.0150 (9)	0.0231 (10)	−0.0012 (7)	−0.0011 (8)	−0.0010 (7)
C18	0.0239 (10)	0.0233 (10)	0.0127 (9)	0.0108 (8)	−0.0020 (7)	−0.0030 (7)
C22	0.0144 (9)	0.0245 (10)	0.0171 (9)	−0.0034 (8)	−0.0022 (7)	−0.0056 (7)
C7	0.0129 (9)	0.0209 (9)	0.0183 (9)	−0.0049 (7)	0.0032 (7)	−0.0080 (7)
C19	0.0206 (10)	0.0170 (9)	0.0113 (9)	0.0027 (7)	−0.0047 (7)	−0.0001 (7)
C9	0.0221 (10)	0.0138 (9)	0.0307 (11)	−0.0008 (8)	0.0029 (8)	−0.0061 (8)
C20	0.0265 (10)	0.0226 (10)	0.0164 (9)	0.0025 (8)	−0.0071 (8)	−0.0097 (7)
C8	0.0193 (10)	0.0206 (10)	0.0249 (10)	−0.0043 (8)	0.0057 (8)	−0.0121 (8)
C3	0.0167 (9)	0.0250 (10)	0.0183 (9)	0.0010 (8)	−0.0031 (7)	0.0000 (8)
C21	0.0255 (11)	0.0230 (10)	0.0242 (10)	−0.0055 (8)	−0.0084 (8)	−0.0082 (8)
C17	0.0149 (9)	0.0244 (10)	0.0168 (9)	0.0086 (8)	0.0036 (7)	0.0030 (7)
C2	0.0206 (10)	0.0162 (9)	0.0268 (10)	0.0016 (8)	−0.0035 (8)	−0.0014 (8)
C6	0.0162 (9)	0.0297 (10)	0.0158 (9)	−0.0030 (8)	0.0006 (7)	−0.0111 (8)
C5	0.0179 (10)	0.0323 (11)	0.0121 (9)	−0.0025 (8)	−0.0020 (7)	−0.0054 (8)
C4	0.0108 (8)	0.0218 (9)	0.0169 (9)	−0.0012 (7)	0.0003 (7)	−0.0017 (7)
C16	0.0124 (9)	0.0183 (9)	0.0152 (9)	0.0042 (7)	−0.0016 (7)	0.0054 (7)
C14	0.0144 (9)	0.0181 (9)	0.0276 (10)	−0.0027 (7)	−0.0079 (8)	0.0033 (8)
O3	0.0323 (9)	0.0276 (8)	0.0520 (10)	−0.0016 (7)	−0.0062 (7)	−0.0190 (7)
O2	0.0472 (12)	0.0437 (10)	0.0732 (13)	−0.0143 (9)	−0.0298 (10)	−0.0008 (10)
O4	0.0711 (15)	0.0491 (11)	0.0621 (13)	−0.0118 (10)	0.0385 (11)	−0.0304 (10)
O1	0.111 (2)	0.0517 (12)	0.0274 (10)	0.0159 (12)	−0.0227 (11)	0.0056 (8)

### Geometric parameters (Å, °)

**Table:**

Cr1—F2	1.8444 (10)	C11—C7	1.401 (3)
Cr1—F1	1.8621 (10)	C10—C9	1.398 (3)
Cr1—N4	2.0566 (15)	C10—H10	0.9501
Cr1—N2	2.0607 (15)	C18—C17	1.367 (3)
Cr1—N3	2.0797 (16)	C18—C19	1.428 (3)
Cr1—N1	2.0934 (15)	C18—H18	0.9501
Cl1—O4	1.4144 (17)	C22—C21	1.404 (3)
Cl1—O1	1.4200 (18)	C22—H22	0.9501
Cl1—O2	1.4310 (19)	C7—C8	1.419 (3)
Cl1—O3	1.4365 (15)	C7—C6	1.429 (3)
N1—C1	1.331 (2)	C19—C20	1.423 (3)
N1—C12	1.363 (2)	C9—C8	1.371 (3)
N3—C13	1.339 (2)	C9—H9	0.9500
N3—C24	1.357 (2)	C20—C21	1.362 (3)
N4—C22	1.335 (2)	C20—H20	0.9500
N4—C23	1.364 (2)	C8—H8	0.9500
N2—C10	1.333 (2)	C3—C2	1.365 (3)
N2—C11	1.359 (2)	C3—C4	1.406 (3)
C12—C4	1.401 (3)	C3—H3	0.9500
C12—C11	1.436 (3)	C21—H21	0.9500
C1—C2	1.404 (3)	C17—C16	1.433 (3)
C1—H1	0.9501	C17—H17	0.9500
C24—C16	1.403 (3)	C2—H2	0.9500
C24—C23	1.436 (2)	C6—C5	1.355 (3)
C23—C19	1.393 (3)	C6—H6	0.9499
C13—C14	1.386 (3)	C5—C4	1.442 (3)
C13—H13	0.9501	C5—H5	0.9500
C15—C14	1.384 (3)	C14—H14	0.9500
C15—C16	1.407 (3)	O5—H5A	1.0444
C15—H15	0.9500	O5—H5B	1.0283
			
?···?	?		
			
F2—Cr1—F1	95.92 (5)	C7—C11—C12	119.83 (16)
F2—Cr1—N4	92.33 (5)	N2—C10—C9	121.87 (17)
F1—Cr1—N4	91.42 (5)	N2—C10—H10	119.1
F2—Cr1—N2	91.83 (6)	C9—C10—H10	119.0
F1—Cr1—N2	91.86 (5)	C17—C18—C19	120.56 (17)
N4—Cr1—N2	174.40 (5)	C17—C18—H18	119.6
F2—Cr1—N3	89.38 (5)	C19—C18—H18	119.9
F1—Cr1—N3	170.08 (5)	N4—C22—C21	121.54 (17)
N4—Cr1—N3	79.95 (6)	N4—C22—H22	119.2
N2—Cr1—N3	96.36 (6)	C21—C22—H22	119.3
F2—Cr1—N1	170.54 (5)	C11—C7—C8	116.47 (17)
F1—Cr1—N1	88.67 (5)	C11—C7—C6	119.10 (17)
N4—Cr1—N1	95.83 (6)	C8—C7—C6	124.39 (17)
N2—Cr1—N1	79.72 (6)	C23—C19—C20	116.89 (17)
N3—Cr1—N1	87.34 (6)	C23—C19—C18	119.31 (17)
O4—Cl1—O1	111.05 (15)	C20—C19—C18	123.80 (17)
O4—Cl1—O2	108.77 (15)	C8—C9—C10	119.92 (17)
O1—Cl1—O2	107.61 (15)	C8—C9—H9	120.0
O4—Cl1—O3	110.09 (11)	C10—C9—H9	120.1
O1—Cl1—O3	110.47 (12)	C21—C20—C19	119.31 (17)
O2—Cl1—O3	108.78 (11)	C21—C20—H20	120.5
C1—N1—C12	118.50 (15)	C19—C20—H20	120.1
C1—N1—Cr1	128.82 (12)	C9—C8—C7	119.61 (18)
C12—N1—Cr1	112.66 (11)	C9—C8—H8	120.3
C13—N3—C24	118.21 (16)	C7—C8—H8	120.1
C13—N3—Cr1	129.08 (13)	C2—C3—C4	119.46 (17)
C24—N3—Cr1	112.64 (12)	C2—C3—H3	120.2
C22—N4—C23	118.54 (16)	C4—C3—H3	120.3
C22—N4—Cr1	127.53 (13)	C20—C21—C22	120.31 (18)
C23—N4—Cr1	113.65 (12)	C20—C21—H21	119.8
C10—N2—C11	118.64 (16)	C22—C21—H21	119.9
C10—N2—Cr1	127.12 (13)	C18—C17—C16	121.10 (17)
C11—N2—Cr1	113.99 (12)	C18—C17—H17	119.4
N1—C12—C4	122.95 (16)	C16—C17—H17	119.6
N1—C12—C11	116.89 (15)	C3—C2—C1	120.06 (17)
C4—C12—C11	120.14 (16)	C3—C2—H2	120.1
N1—C1—C2	121.76 (17)	C1—C2—H2	119.8
N1—C1—H1	119.1	C5—C6—C7	121.37 (17)
C2—C1—H1	119.2	C5—C6—H6	119.2
N3—C24—C16	123.29 (17)	C7—C6—H6	119.5
N3—C24—C23	117.20 (16)	C6—C5—C4	120.78 (17)
C16—C24—C23	119.51 (17)	C6—C5—H5	119.7
N4—C23—C19	123.39 (16)	C4—C5—H5	119.5
N4—C23—C24	116.13 (16)	C12—C4—C3	117.27 (17)
C19—C23—C24	120.48 (17)	C12—C4—C5	118.77 (17)
N3—C13—C14	122.54 (18)	C3—C4—C5	123.94 (17)
N3—C13—H13	118.9	C24—C16—C15	116.91 (17)
C14—C13—H13	118.6	C24—C16—C17	119.01 (17)
C14—C15—C16	119.58 (17)	C15—C16—C17	124.08 (17)
C14—C15—H15	120.1	C15—C14—C13	119.45 (17)
C16—C15—H15	120.3	C15—C14—H14	120.1
N2—C11—C7	123.46 (17)	C13—C14—H14	120.4
N2—C11—C12	116.68 (16)	H5A—O5—H5B	101.7
			
?—?—?—?	?		

### Hydrogen-bond geometry (Å, °)

**Table:**

*D*—H···*A*	*D*—H	H···*A*	*D*···*A*	*D*—H···*A*
O5—H5B···F1^i^	1.03	1.69	2.7183 (19)	175

Symmetry codes: (i) *x*−1, *y*, *z*.
